# Comparative prognostic analysis of allogeneic hematopoietic stem cell transplantation regimens in the treatment of severe aplastic anemia

**DOI:** 10.3389/fimmu.2026.1936533

**Published:** 2026-10-05

**Authors:** Ting Lin, Xianlin Chen, Xiaohong Yuan, Zhijuan Zhu, Nainong Li, Xiaofan Li

**Affiliations:** 1 Fujian Institute of Hematology, Fujian Provincial Key Laboratory on Hematology,FUjian Medical University Union Hospital, Fuzhou, China; 2 Translational Medicine Center on Hematology, Fujian Medical University, Fuzhou, China

**Keywords:** allogeneic hematopoietic stem cell transplantation, aplastic anemia, conditioning regimen, haploidentical transplantation, matched sibling donor

## Abstract

**Background:**

Severe aplastic anemia (SAA) is a fatal bone marrow failure disorder. Allogeneic hematopoietic stem cell transplantation (allo-HSCT) is the definitive curative treatment for SAA. This study evaluated the prognosis of patients with severe aplastic anemia (SAA) receiving different allo-HSCT regimens.

**Methods:**

A retrospective analysis was performed on 62 patients with aplastic anemia who underwent allo-HSCT between January 2015 and August 2024. The cohort comprised 32 males and 30 females, with a median age of 12 years (range, 1.5–44 years) at transplantation; 28 patients were ≥14 years old and 34 were <14 years. Forty-eight patients were diagnosed with severe AA (SAA) and 14 with very severe AA (VSAA). Twenty-two patients underwent HLA-matched sibling donor transplantation(MSD-HSCT), and 40 underwent HLA-haploidentical sibling donor transplantation(Haplo-HSCT).Three conditioning regimens were administered: cyclophosphamide plus anti-thymocyte globulin (CY+ATG, CA, n=11), fludarabine + cyclophosphamide + ATG (Flu+CY+ATG, FCA, n=26), and fludarabine + melphalan + ATG (Flu+Mel+ATG, FMA, n=25).

**Results:**

The median follow-up duration of the 62 patients was 38 months (range: 0–136 months). Twelve deaths occurred during follow-up. Post-transplant complications included post-transplant lymphoproliferative disorder (PTLD) in 3 patients (4.84%), acute graft-versus-host disease (aGVHD) in 7 patients (11.29%), chronic GVHD (cGVHD) in 2 patients (3.23%), Epstein-Barr virus (EBV) viremia in 10 patients (16.13%), and invasive fungal disease in 1 patient (1.62%). The median time to neutrophil engraftment was 13 days (evaluable in 55 patients). Kaplan-Meier survival analysis revealed no statistically significant difference in 5-year overall survival (OS) between MSD-HSCT and Haplo-HSCT groups (P = 0.582). The 5-year OS rate was 76.8% for the MSD-HSCT (n=22) and 80.6% for the Haplo-HSCT group (n=40). No significant intergroup difference in 5-year OS was detected across the three conditioning regimens (P = 0.85); the 5-year OS rates of the CA, FCA, and FMA groups were 81.6%, 76.8%, and 84.0%, respectively.

**Conclusions:**

(1)MSD-HSCT and Haplo-HSCT yield comparable 5-year OS. Haploidentical related donors serve as a reliable alternative when fully matched donors are unavailable. (2) No statistically significant difference in 5-year overall survival (OS) was observed among the CA, FCA, and FMA conditioning regimens. (3) the FMA regimen may be considered as a favorable alternative to avoid the cyclophosphamide related toxicities.

## 1 Background

Aplastic anemia (AA) constitutes a heterogeneous syndrome of bone marrow failure induced by multiple etiologies, characterized by profound depletion of hematopoietic stem and progenitor cells, hypocellular bone marrow, and pancytopenia in peripheral blood. This disease predominantly affects children, young adults, and individuals older than 60 years (1, 2). Diagnostic stratification of acquired aplastic anemia was performed based on the modified Camitta criteria. Patients were diagnosed with AA when presenting with at least two of the following peripheral blood abnormalities (Hb < 100 g/L, PLT < 50 × 10^9^/L, ANC < 1.5 × 10^9^/L) after exclusion of other causes of bone marrow failure. SAA was defined as bone marrow cellularity < 25% (or 25%–50% with < 30% residual hematopoietic cells), accompanied by at least two of the following: ANC < 0.5 × 10^9^/L, PLT < 20 × 10^9^/L, and absolute reticulocyte count < 20 × 10^9^/L. VSAA was defined as SAA with an ANC < 0.2 × 10^9^/L (3).The core clinical manifestations include recurrent infection, hemorrhage, and progressive anemia. For newly diagnosed AA patients, first-line therapeutic strategies are determined by disease severity, donor availability, and patient age; standard initial interventions consist of either allo-HSCT or immunosuppressive therapy (IST) (4). Outcomes of allo-HSCT for AA have improved remarkably over the past decade, with long-term survival rates ranging from 70% to 90%.Currently, most allo-HSCT protocols for SAA adopt cyclophosphamide-based conditioning regimens combined with ATG (5). Nevertheless, cyclophosphamide carries prominent cardiac toxicities and causes irreversible impairment of growth and reproductive function in children. In addition, the development of intensified GVHD prophylaxis strategies breaks the restriction of HLA matching. Clinical outcomes of the haplo-HSCT for SAA have continuously improved. To compare the efficacy of allo-HSCT stratified by distinct conditioning regimens and varying HLA matching degrees between donors and recipients, this retrospective study analyzes clinical data from 62 AA patients who received allo-HSCT at the Hematopoietic Stem Cell Transplant Center of Fujian Medical University Union Hospital from January 2015 to August 2024.

## 2 Patients and methods

### 2.1 Study population

This retrospective study enrolled 62 patients diagnosed with AA who received allo-HSCT between January 2015 and August 2024 at the Hematopoietic Stem Cell Transplant Center of Fujian Medical University Union Hospital. Clinical data were collected and analyzed retrospectively. All participants provided written informed consent before transplantation.

### 2.2 Conditioning regimens and GVHD prophylaxis

Eleven patients received the CA conditioning regimen: cyclophosphamide (50 mg/kg per day, days −5 to −2) plus rabbit ATG (2.5 mg/kg per day, days −5 to −2). Twenty-six patients were treated with the FCA regimen: fludarabine (30 mg/m² per day, days −5 to −2), cyclophosphamide (50 mg/kg per day, days −5 to −2), and rabbit ATG (2.5 mg/kg per day, days −5 to −2). Twenty-five patients received the FMA regimen: fludarabine (30 mg/m² per day, days −5 to −2), melphalan (60 mg/m² per day for 2 days, days −3 to −2), and rabbit ATG (2.5 mg/kg per day, days −5 to −2).All patients received cyclosporine A (CsA) combined with methotrexate (MTX) and mycophenolate mofetil (MMF) for GVHD prophylaxis.

### 2.3 Supportive care

All patients were hospitalized in laminar flow wards until neutrophil recovery after transplantation. Rituximab was administered to eliminate donor-specific antibodies (DSA) in patients with positive pre-transplant DSA. Trimethoprim-sulfamethoxazole was orally administered before transplantation. Posaconazole oral suspension was initiated on post-transplant day 1 for primary antifungal prophylaxis and discontinued at 6 months after transplantation. Platelet transfusions were administered when platelet counts dropped below 20×10^9^/L or active bleeding occurred. Red blood cell transfusions were given for hemoglobin levels lower than 60 g/L. Granulocyte colony-stimulating factor (G-CSF) was administered starting on post-transplant day 6 until absolute neutrophil count (ANC) returned to normal.

Perianal swabs for carbapenem-resistant Enterobacteriaceae (CRE) detection were collected twice weekly during hospitalization to guide antibiotic adjustment. Cytomegalovirus (CMV) and EBV monitoring were performed twice weekly during admission and once weekly after discharge.

### 2.4 Diagnostic criteria and definitions

All AA diagnoses followed the Chinese Expert Consensus on the Diagnosis and Treatment of Aplastic Anemia (2017 Edition). Diagnosis and grading criteria for aGVHD complied with the 2020 European Society for Blood and Marrow Transplantation (EBMT) consensus (6, 7). Neutrophil engraftment was defined as a sustained ANC ≥0.5×10^9^/L for three consecutive days. Platelet engraftment was defined as platelet count ≥20×10^9^/L for seven consecutive days without platelet transfusion support.

### 2.5 Follow-up

Survival data were collected via review of inpatient and outpatient medical records combined with telephone follow-up. The cutoff date for follow-up was June 8, 2026. The median follow-up time after transplantation was 38 months (range: 0–136 months). The primary observational endpoint was OS, defined as the interval from transplantation to all-cause death or the last follow-up visit.

### 2.6 Statistical analysis

Statistical analyses were conducted using SPSS 24.0 software. Categorical variables were compared using the chi-square test or Fisher’s exact test as appropriate. Continuous variables were compared with independent t-tests for normally distributed data or Mann-Whitney U tests for non-normal data. Kaplan-Meier curves were generated to calculate cumulative survival rates, and the Log-rank test was used for intergroup survival comparisons. A two-tailed P value <0.05 indicated statistically significant differences. Cumulative incidence functions for post-transplant complications were estimated using the Aalen-Johansen competing-risk method, where death in the absence of the respective complication was defined as the competing event. The 95% confidence intervals (CIs) were generated by 5000-fold bootstrap resampling. To mitigate baseline imbalances between the FMA and FCA groups, 1:1 optimal propensity-score matching (PSM) was performed as the primary adjusted analysis, and stabilized inverse-probability-of-treatment weighting (IPTW) was further applied as sensitivity analysis. Given the relatively small sample size of the CA group (n=11), patients receiving CA conditioning were not included in the PSM-IPTW adjustment. Eight baseline covariates were incorporated to calculate propensity scores, including age at transplantation, sex, diagnosis (SAA versus VSAA), pre-transplant immunosuppressive therapy, EBMT risk score ≥2, interval from diagnosis to transplantation ≥1 year, HLA matching status, and ABO compatibility. After PSM, weighted Kaplan-Meier and weighted Cox proportional-hazards regression were performed for survival outcome analysis. The proportional-hazards assumption was validated by Schoenfeld residual tests. Standardized mean differences (SMD) were calculated to evaluate covariate balance before and after adjustment. An SMD < 0.1 indicated satisfactory balance between groups.

## 3 Results

### 3.1 Baseline patient characteristics

Sixty-two patients met the inclusion criteria of this study. Baseline characteristics of patients and donors were summarized in **Table 1**. The median patient age was 12 years (range: 1.5–44 years), including 32 males and 30 females. Forty-eight patients had SAA, 14 had VSAA. Sixteen patients received combined peripheral blood plus bone marrow grafts, and 46 received peripheral blood stem cell grafts. Twenty-two patients received MSD-HSCT and 40 received Haplo-HSCT. The number of patients receiving FCA, FMA and CA conditioning regimens were 26, 25 and 11, respectively. Twenty-nine donor-recipient pairs had ABO blood type incompatibility, and 33 pairs had ABO compatibility. Thirty-five patients had an EBMT risk score ≥2, and 27 patients had a score <2. Sixteen patients were transplanted more than 1 year after diagnosis, and 46 patients received transplantation within 1 year of diagnosis. Thirty-one patients received immunosuppressive agents before transplantation, and 31 patients did not. **Table 1** summarized the baseline characteristics of all 62 AA patients undergoing allo-HSCT, and **Table 2** listed clinical data stratified by three different conditioning regimens.

**Table 1:** Baseline characteristics of 62 AA patients undergoing allogeneic hematopoietic stem cell transplantation.

Variable	Total (n=62)	MSD (n=22)	Haplo-HSCT (n=40)	P value
Age at diagnosis [years, median (range)]	12 (1.5-44)	15 (5-44)	9.5 (1.5-38)	0.013
Gender, n (%)				0.650
Male	32 (51.61)	10 (45.45)	22 (55.00)	
Female	30 (48.39)	12 (54.55)	18 (45.00)	
Diagnosis, n (%)				0.539
SAA	48 (77.42)	16 (72.73)	32 (80.00)	
VSAA	14 (22.58)	6 (27.27)	8 (20.00)	
Graft source, n (%)				0.002
BM+PBS	16 (25.81)	0 (0.00)	16 (40.00)	
PBS	46 (74.19)	22 (100.00)	24 (60.00)	
Conditioning regimen, n (%)				<0.001
CA	11 (17.74)	11 (50.00)	0 (0.00)	
FCA	26 (41.94)	3 (13.64)	23 (57.50)	
FMA	25 (40.32)	8 (36.36)	17 (42.50)	
Donor-recipient ABO matching status, n (%)				0.138
Matched	33 (53.23)	15 (68.18)	18 (45.00)	
Mismatched	29 (46.77)	7 (31.82)	22 (55.00)	
EBMT score, n (%)				0.099
≥2	35 (56.45)	16 (72.73)	19 (47.50)	
<2	27 (43.55)	6 (27.27)	21 (52.50)	
Pre-transplant IST, n (%)				1.000
Yes	31 (50.00)	11 (50.00)	20 (50.00)	
No	31 (50.00)	11 (50.00)	20 (50.00)	
Interval from diagnosis to transplantation ≥1 year, n (%)				0.618
Yes	16 (25.81)	7 (31.82)	9 (22.50)	
No	46 (74.19)	15 (68.18)	31 (77.50)	

AA, aplastic anemia; MSD, matched sibling donor; Haplo-HSCT, haploidentical hematopoietic stem cell transplantation; SAA, severe aplastic anemia; VSAA, very severe aplastic anemia; BM, bone marrow; PBS, peripheral blood stem cell; CA, cyclophosphamide + antithymocyte globulin; FCA, fludarabine + cyclophosphamide + antithymocyte globulin; FMA, fludarabine + melphalan + antithymocyte globulin; EBMT, European Society for Blood and Marrow Transplantation; IST, immunosuppressive therapy.

**Table 2:** Baseline characteristics of AA patients treated with different allo-HSCT conditioning regimens.

Variable	CA (n=11)	FCA (n=26)	FMA (n=25)	P value
Age at diagnosis [years, median (range)]	14.5 (5-39)	9 (1.5-38)	14 (2-44)	0.042
Gender, n (%)				0.412
Male	5 (45.45)	16 (61.54)	11 (44.00)	
Female	6 (54.55)	10 (38.46)	14 (56.00)	
Diagnosis, n (%)				0.037
SAA	6 (54.55)	19 (73.08)	23 (92.00)	
VSAA	5 (45.45)	7 (26.92)	2 (8.00)	
Graft source, n (%)				<0.001
BM+PBS	0 (0.00)	16 (61.54)	0 (0.00)	
PBS	11 (100.00)	10 (38.46)	25 (100.00)	
HLA matching degree, n (%)				<0.001
MSD	11 (100.00)	3 (11.54)	8 (32.00)	
Haplo-HSCT	0 (0.00)	23 (88.46)	17 (68.00)	
Donor-recipient ABO matching status, n (%)				0.298
Matched	6 (54.55)	11 (42.31)	16 (64.00)	
Mismatched	5 (45.45)	15 (57.69)	9 (36.00)	
EBMT score, n (%)				0.859
≥2	7 (63.64)	14 (53.85)	14 (56.00)	
<2	4 (36.36)	12 (46.15)	11 (44.00)	
Pre-transplant IST, n (%)				0.937
Yes	5 (45.45)	13 (50.00)	13 (52.00)	
No	6 (54.55)	13 (50.00)	12 (48.00)	
Interval from diagnosis to transplantation ≥1 year, n (%)				0.401
Yes	2 (18.18)	9 (34.62)	5 (20.00)	
No	9 (81.82)	17 (65.38)	20 (80.00)	

AA, aplastic anemia; allo-HSCT, allogeneic hematopoietic stem cell transplantation; CA, cyclophosphamide + antithymocyte globulin; FCA, fludarabine + cyclophosphamide + antithymocyte globulin; FMA, fludarabine + melphalan + antithymocyte globulin; SAA, severe aplastic anemia; VSAA, very severe aplastic anemia; BM, bone marrow; PBS, peripheral blood stem cell; MSD, matched sibling donor; Haplo-HSCT, haploidentical hematopoietic stem cell transplantation; EBMT, European Society for Blood and Marrow Transplantation; IST, immunosuppressive therapy.

### 3.2 Hematopoietic reconstitution

Twelve deaths occurred during follow-up, including 8 deaths from uncontrolled severe infection and 4 deaths from severe aGVHD. Seven patients died before neutrophil engraftment; the median time to neutrophil recovery among the remaining 55 evaluable patients was 13 days (range: 9–24 days). Nine patients died prior to platelet engraftment; the median platelet engraftment time of the other 53 patients was 16 days (range: 10–35 days).

### 3.3 Post-transplant complications

Seven patients developed aGVHD. The cumulative incidence of aGVHD was 8.06% (95% CI, 3.2-16.1%) at day 100 post-transplantation, 11.29% (95% CI, 4.8-21.0%) at 1-year post-transplantation, and 11.29% (95% CI, 4.8-19.4%) at last follow-up. Two patients developed cGVHD. The 1-year cumulative incidence of cGVHD was 1.61% (95% CI, 0-4.8%), and the overall cumulative incidence was 3.23% (95% CI, 0-8.1%) at last follow-up. Four patients experienced CMV infection, with a 1-year cumulative incidence of 6.45% (95% CI, 1.6-14.5%) and an overall cumulative incidence of 6.45% (95% CI, 1.6-12.9%) at last follow-up. Ten patients developed EBV infection. Its 1-year cumulative incidence was 11.29% (95% CI, 4.8-21.0%), and the overall cumulative incidence reached 16.52% (95% CI, 8.1-26.8%) at last follow-up. Hemorrhagic cystitis occurred in two patients, with a 1-year cumulative incidence of 1.61% (95% CI, 0-4.8%) and an overall cumulative incidence of 3.23% (95% CI, 0-8.1%) at last follow-up. Three patients developed post-transplant lymphoproliferative disorder (PTLD). The 1-year cumulative incidence of PTLD was 1.61% (95% CI, 0-4.8%), and the overall cumulative incidence was 4.84% (95% CI, 0-11.3%) at last follow-up. One patient developed herpes zoster, with both 1-year and final cumulative incidences of 1.61% (95% CI, 0-4.8%). One patient suffered from invasive fungal disease, with both 1-year and final cumulative incidences of 1.61% (95% CI, 0-4.8%).Hematopoietic reconstitution, mortality, and complications in AA patients treated with different conditioning regimens are summarized in **Table 3**.

**Table 3:** Hematopoietic reconstitution, mortality, and complications in AA patients treated with different conditioning regimens.

Variable	CA (n=11)	FCA (n=26)	FMA (n=25)	P value
Hematopoietic reconstitution				
Neutrophil engraftment, n (%)	10 (90.91)	24 (92.31)	21 (84.00)	0.624
Median time to neutrophil engraftment, d (range)	13 (9-24)	13 (11-21)	13 (10-17)	0.840
Platelet engraftment, n (%)	10 (90.91)	22 (84.62)	21 (84.00)	0.852
Median time to platelet engraftment, d (range)	15 (11-35)	17 (12-33)	16 (10-30)	0.551
Death, n (%)	2 (18.18)	6 (23.08)	4 (16.00)	0.810
GVHD				
aGVHD, n (%)	0 (0.00)	4 (15.38)	3 (12.00)	0.397
cGVHD, n (%)	0 (0.00)	1 (3.85)	1 (4.00)	0.800
Infectious complications				
EBV infection, n (%)	1 (9.09)	2 (7.69)	7 (28.00)	0.112
EBV-related PTLD, n (%)	0 (0.00)	2 (7.69)	0 (0.00)	0.239
CMV infection, n (%)	0 (0.00)	2 (7.69)	2 (8.00)	0.630
Hemorrhagic cystitis, n (%)	0 (0.00)	1 (3.85)	1 (4.00)	0.800
Herpes zoster, n (%)	0 (0.00)	1 (3.85)	0 (0.00)	0.495
IFD, n (%)	1 (9.09)	0 (0.00)	0 (0.00)	0.095

AA, aplastic anemia; CA, cyclophosphamide + antithymocyte globulin; FCA, fludarabine + cyclophosphamide + antithymocyte globulin; FMA, fludarabine + melphalan + antithymocyte globulin; aGVHD, acute graft-versus-host disease; cGVHD, chronic graft-versus-host disease; EBV, Epstein-Barr virus; PTLD, post-transplant lymphoproliferative disorder; CMV, cytomegalovirus; IFD, invasive fungal disease.

### 3.4 Survival outcomes

The estimated 5-year OS rate was 76.8% in the MSD-HSCT group and 80.6% in the Haplo-HSCT group(p=0.582) (**Figure 1**). The 5-year OS rates of the CA, FCA and FMA conditioning groups were 81.6%, 76.8%, and 84.0%, respectively(p=0.85) (**Figure 2**).

**Figure 1:**
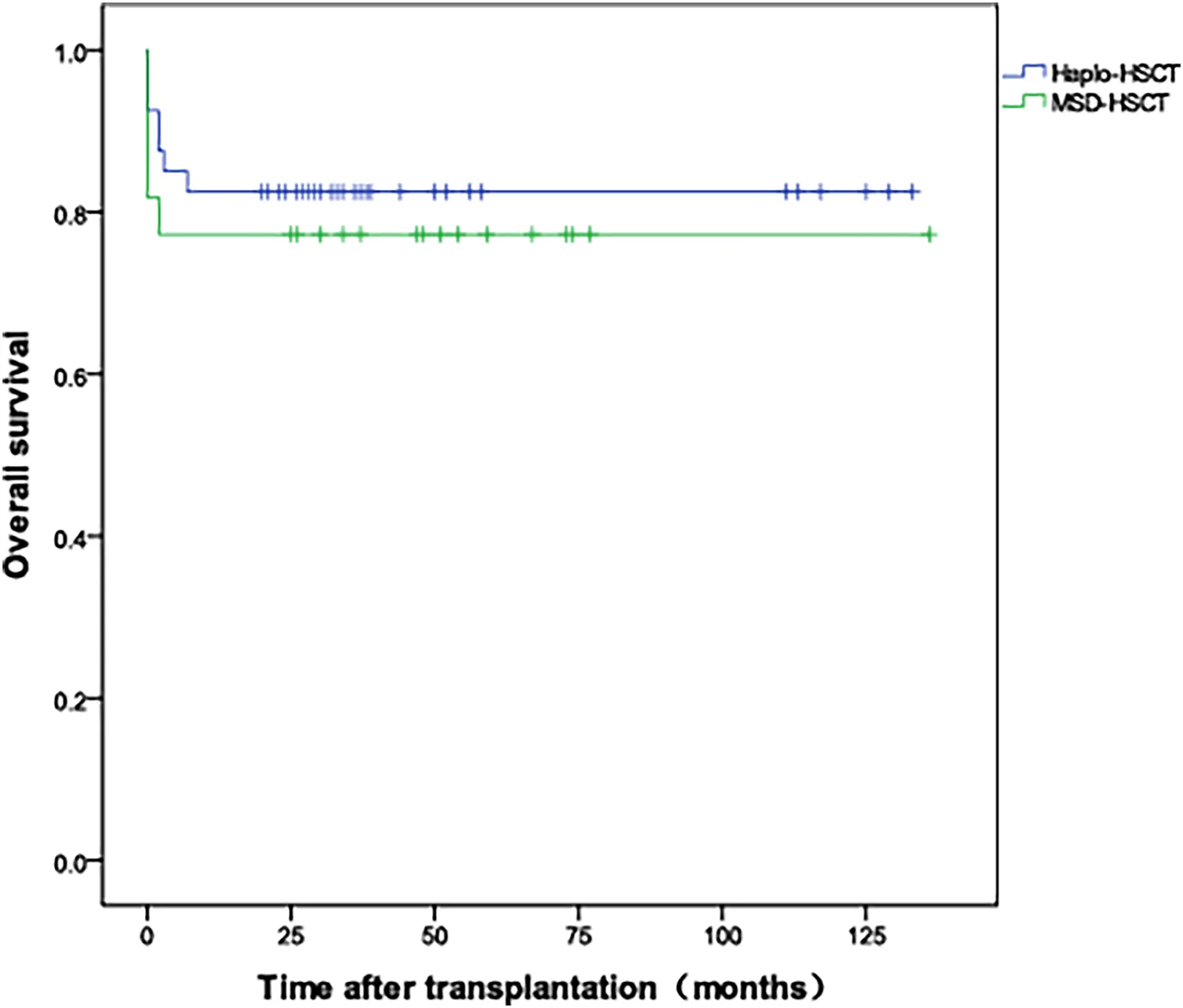
Overall survival of 62 patients with aplastic anemia following allo-HSCT stratified by donor source: haploidentical-HSCT versus matched-sibling donor HSCT.

**Figure 2:**
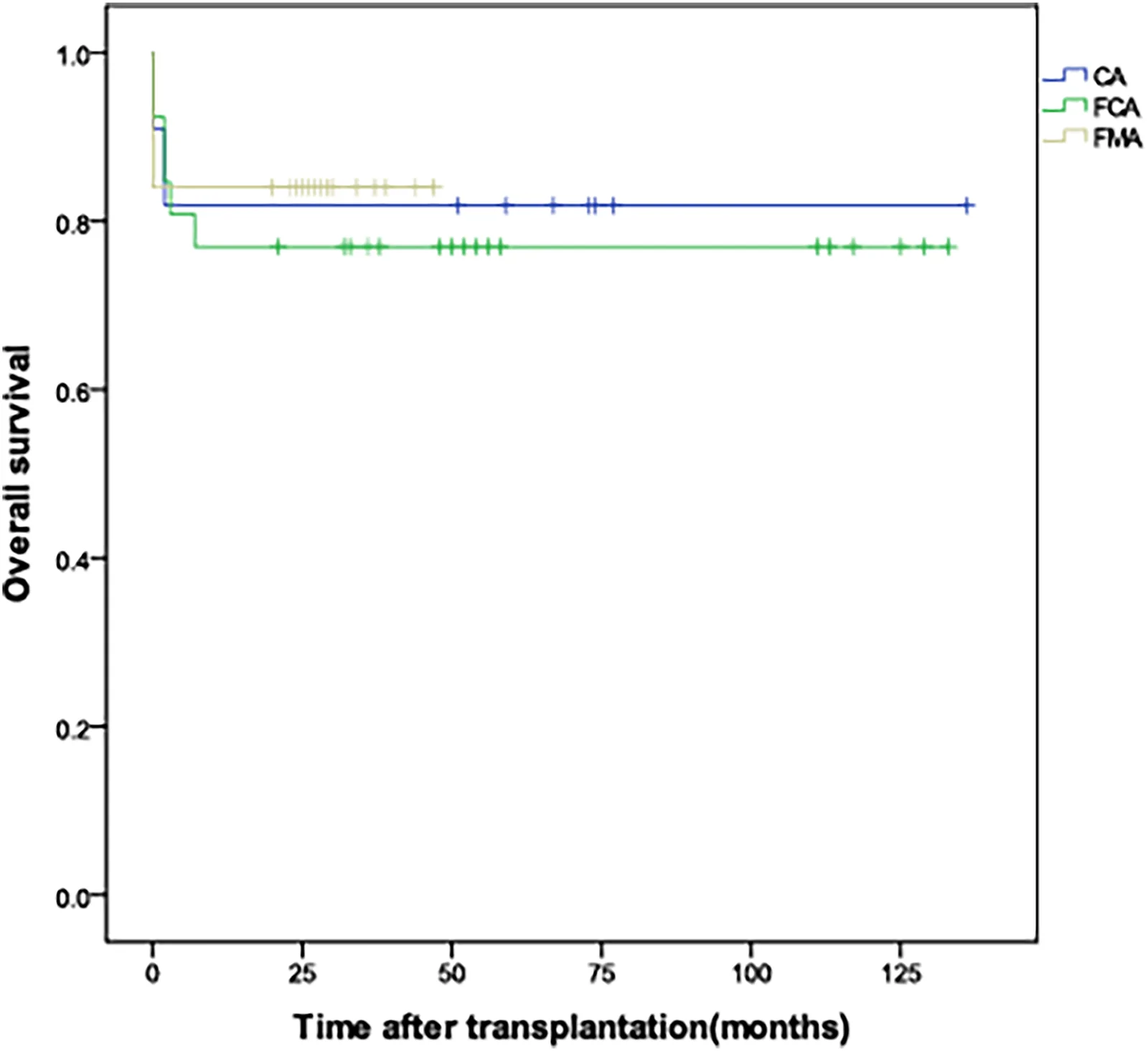
Overall survival curves for 62 patients with aplastic anemia who received allo-HSCT with three different conditioning regimens.

Given prominent baseline imbalances between FMA and FCA groups, we further performed propensity-score-based adjustment restricted to these two cohorts. After 1:1 optimal propensity-score matching, 16 well-balanced pairs (32 patients) were generated. In the matched cohort, numerically superior overall survival was observed in the FMA group (3-year OS 93.8%) compared with the FCA group (3-year OS 75.0%), without statistical significance (log-rank p=0.156; HR = 0.236, 95%CI 0.026-2.115, P = 0.197). Stabilized-IPTW sensitivity analysis including all original 51 FMA/FCA patients yielded consistent results, showing a borderline-lower mortality risk for FMA (HR = 0.271, 95%CI 0.061-1.204, P = 0.087). Hazard ratios were consistently below 1 across both PSM and IPTW analyses, suggesting an observational numerical trend toward better survival for FMA, although no statistically significant benefit was demonstrated.

Baseline covariate balance before and after propensity-score adjustment is shown in **Supplementary Figures S1**, **S2**; **Supplementary Tables S1**, **S2.** Kaplan-Meier survival curves for the PSM-matched cohort are presented in **Supplementary Figure S3**. Hazard ratios derived from PSM-matched and stabilized-IPTW analyses are visualized in **Supplementary Figure S4**, and detailed survival outcomes are summarized in **Supplementary Table S3**.

## 4 Discussion

Haploidentical HSCT has emerged as a viable alternative transplant modality over the past decade, owing to advances in supportive care, standardized ATG application, and optimized donor selection, which fulfills the goal of universal donor accessibility for patients without fully matched siblings. A previous study by ElGohary G et al. reported that 97.3% of patients achieved durable engraftment after haploidentical HSCT for AA, with acceptable rates of regimen-related toxicity (RRT, 6.2%) and transplant-related mortality (TRM, 6.7%). Approximately one-quarter of patients developed grade II–IV aGVHD (26.5%) or cGVHD (25.0%). These data confirm robust engraftment rates and manageable RRT, TRM and GVHD risks for most patients with SAA or VSAA (8). Single-center retrospective study by Zhang et al. enrolled 76 SAA patients undergoing haplo-HSCT. Three patients experienced early mortality, and the overall survival (OS) reached (78.6 ± 5.0)%. Notably, patients aged ≤25 years achieved a 5-year OS of (86.5 ± 4.8)%, with comparable outcomes to those receiving MSD-HSCT (9). Xu ZL et al. illustrated that optimized intensified GVHD prophylaxis strategies greatly improved the performance of haploidentical SCT for SAA, with engraftment rates exceeding 90% and severe GVHD incidence lower than 10% (10). Two mainstream GVHD prophylaxis strategies, G-CSF/ATG and post-transplant cyclophosphamide (PTCy)-based regimens, carry distinct advantages and limitations (11). Ma X et al. developed a modified G-CSF/ATG/low-dose PTCy prophylaxis protocol that yielded encouraging outcomes in SAA patients receiving haploidentical HSCT, including 100% primary engraftment, mild toxicities, reduced GVHD incidence, and 100% OS and graft-failure-free survival (GFFS). This protocol maximizes engraftment probability while minimizing GVHD risk (12). Haploidentical HSCT based on G-CSF and ATG yields survival outcomes equivalent to MSD transplantation for SAA, with graft-failure-free survival (FFS) exceeding 80% in both upfront and salvage transplant cohorts. Consequently, G-CSF/ATG-based haploidentical transplant protocols are widely adopted across global transplant centers (10). Our center also utilizes G-CSF combined with ATG to mitigate GVHD and overcome inherent drawbacks of haploidentical transplantation. Consistent with previous literature, our results demonstrate comparable long-term survival: The 5-year OS rate was 76.8% for the MSD-HSCT and 80.6% for the Haplo-HSCT group.

Bejanyan N et al. summarized mainstream conditioning regimens for SAA bone marrow transplantation. Flu/Cy/ATG and Cy/ATG represent standard protocols for MSD transplantation. Flu/Cy/ATG is more commonly administered to older patients, while Cy/ATG is preferred for younger individuals, yet both regimens produce excellent survival rates (93% and 91%, respectively), independent of patient age. For unrelated donor transplantation, ATG/Cy plus low-dose total body irradiation (TBI, 200 cGy) and Flu/ATG/Cy plus TBI 200 cGy serve as primary conditioning options with equivalent survival benefits. Graft failure rates remain low for both Flu/Cy/ATG and Cy/ATG protocols (13). One prior study augmented Cy+ATG conditioning with fludarabine and busulfan to intensify immunosuppression, aiming to eradicate residual host hematopoiesis and reduce graft rejection risk. All 22 enrolled SAA patients achieved rapid and sustained hematopoietic reconstitution after allo-HSCT; median neutrophil and platelet engraftment times were 15 days (range: 11–22 days) and 16 days (range: 12–27 days), respectively. Three patients died of infection or severe GVHD, while all surviving patients maintained persistent donor hematopoiesis without disease relapse. The 2-year cumulative survival rate reached 86.4%, and all survivors maintained satisfactory quality of life (14).Ashizawa M et al. followed 13 patients receiving a conditioning regimen consisting of fludarabine, reduced-dose Cy and ATG. All patients except one early death achieved full donor engraftment, with a 5-year survival rate of 76.9%. The incidences of acute and chronic GVHD were consistent with historical reports of fludarabine-based conditioning (15, 16). In a prospective phase 2 trial of upfront HLA-haploidentical BMT with PTCy for acquired SAA, DeZern et al. reported a 3-year OS of 92% (95% CI, 83–100%), with only 7% grade II–IV acute GVHD and 4% chronic GVHD. A dose-finding analysis revealed that 400 cGy TBI (vs 200 cGy) eliminated graft failure (0/20 vs 3/7; P = 0.01) and achieved 100% OS in 20 consecutive patients (17).Nevertheless, a study of 88 patients reported a graft failure rate as high as 17% when pediatric patients received Flu plus low-dose Cy and ATG, while adult patients received the same regimen combined with 2 Gy TBI (18). Another cohort of 50 patients receiving Flu/Cy plus alemtuzumab exhibited a 12% graft failure rate (16). To optimize Cy dosage and reduce graft rejection risk, the Blood and Marrow Transplant Clinical Trials Network (BMT CTN) conducted a prospective trial enrolling 96 AA patients comparing four Cy doses (0, 50, 100, and 150 mg/kg). Subtherapeutic Cy doses lead to high primary graft failure, whereas excessive doses trigger severe toxicities. High-dose cyclophosphamide is well-documented to induce cardiac toxicity and hemorrhagic cystitis (19, 20). Current guidelines recommend Cy doses ranging from 50 to 100 mg/kg. However, graft failure (primarily late graft failure) remains prevalent, highlighting the urgent clinical need to develop cyclophosphamide-free conditioning backbones (21–23).

Yoshida N et al. reported outstanding outcomes with Flu/melphalan-based conditioning regimens, with OS and event-free survival (EFS) approaching 100%, late graft failure reduced to 3%, and no primary graft failure or donor-type aplastic anemia observed. For patients with acquired bone marrow failure, the 3-year OS and EFS reached 96% and 94%, respectively, while both endpoints achieved 100% in patients with refractory cytopenia. These findings support Flu/melphalan-based conditioning as an optimal strategy for bone marrow failure syndromes (24). Japanese clinical guidelines recommend a conditioning regimen containing fludarabine (125 mg/m²), melphalan (140 mg/m²), and ATG (5 mg/kg) (with or without low-dose TBI) for pediatric patients with acquired bone marrow failure undergoing allo-HSCT (25). A domestic retrospective study from the 920th Hospital enrolled 20 AA patients receiving haploidentical HSCT with melphalan-containing conditioning between 2020 and 2021. With a median follow-up of 307 days, the overall GVHD incidence was only 5%, and both OS and EFS reached 95% (26).Our study detected no statistically significant intergroup difference in 5-year OS across CA, FCA and FMA regimens (P=0.85), with corresponding 5-year OS rates of 81.6%, 76.8%, and 84.0%. To account for baseline imbalances between FMA and FCA groups, we performed 1:1 optimal propensity-score -matching as primary analysis and stabilized-IPTW as sensitivity analysis. Consistent hazard-ratio directions were observed in both adjusted analyses, indicating a numerical trend toward favorable survival for patients receiving FMA conditioning. Importantly, even after comprehensive confounder adjustment using these two statistical approaches, the small sample size and limited number of clinical events preclude us from drawing definitive conclusions regarding the superiority of one conditioning regimen over the other. Residual unmeasured confounding cannot be fully excluded despite statistical adjustment, and this constitutes the major limitation of the present study.

Melphalan may avoid the irreversible long term damage to childhood growth and reproductive function induced by cyclophosphamide. Clinical reports confirm female patients exposed to melphalan doses up to 240 mg/m^2^ retain normal fertility and successful pregnancy (27). Another cohort demonstrated regular spontaneous menstruation and normal gonadotropin/sex hormone levels in post pubertal females receiving Flu/melphalan conditioning (28). Notably, we observed in our clinical practice that a proportion of female patients who received the FMA conditioning regimen retained menstrual function after transplantation. Nevertheless, considering the lack of statistically-proven survival benefit in our cohort, we still cannot strongly recommend FMA as a preferential regimen. The potential gonadal sparing property of FMA warrants further validation in larger scale, multicenter external cohorts.

## 5 Conclusions

The clinical application of haploidentical HSCT for SAA continues to expand, and this modality represents a reliable alternative for patients lacking MSD. No statistically significant difference in overall survival was found among the FMA, FCA, and CA conditioning regimens. Although a numerical survival trend favoring FMA was observed after propensity-score-based adjustment, definitive survival superiority could not be established. FMA might represent a potential option to avoid cyclophosphamide-related toxicities including cardiotoxicity, gonadotoxicity, and hemorrhagic cystitis.

Nearly all current relevant studies rely on retrospective cohort data, which carry inherent limitations including selection bias and limited sample size. Further larger-scale multicenter retrospective or prospective clinical trials are urgently required to validate our findings, identify optimal conditioning backbones, standardize graft-source selection, and refine GVHD prophylaxis strategies, to further improve long-term survival and quality of life for SAA patients.

## Data Availability

The raw data supporting the conclusions of this article will be made available by the authors, without undue reservation.
