# Supplementary material for: Comparative prognostic analysis of allogeneic hematopoietic stem cell transplantation regimens in the treatment of severe aplastic anemia
Source: Front Immunol. 2026 Oct 5;17:1936533. doi: 10.3389/fimmu.2026.1936533 (PMC13677466; doi:10.3389/fimmu.2026.1936533)
Supplement: Supplementary file 1 [file DataSheet1.docx]

**Supplementary Table S1. Baseline characteristics of FMA and FCA patients before and after 1:1 optimal propensity-score matching**

Continuous variables presented as median (range); categorical variables as n (%). SMD, standardized mean difference; SMD < 0.1 indicates acceptable balance.

| **Covariate** | **Pre-matching cohort** | | **SMD(Pre-match)** | **Post-matching cohort** | | **SMD(Post-match)** |
| --- | --- | --- | --- | --- | --- | --- |
|  | **FMA (n=25)** | **FCA (n=26)** |  | **FMA (n=16)** | **FCA (n=16)** |  |
| Age at transplantation, years | 18.52 (2-44) | 13.02 (1.5-38) | 0.481 | 15.1 (2-42) | 14.3 (1.5-38) | 0.172 |
| Sex, female, n (%) | 14 (56.0) | 10 (38.5) | 0.355 | 8 (50.0) | 7 (43.8) | 0.125 |
| Diagnosis VSAA, n (%) | 2 (8.0) | 7 (26.9) | 0.505 | 2 (12.5) | 3 (18.8) | 0.174 |
| Pre-transplant IST-Yes, n (%) | 13 (52.0) | 13 (50.0) | 0.040 | 9 (56.3) | 8 (50.0) | 0.126 |
| EBMT score ≥2, n (%) | 14 (56.0) | 14 (53.8) | 0.044 | 9 (56.3) | 8 (50.0) | 0.126 |
| Diagnosis-to-transplant ≥1y, n (%) | 5 (20.0) | 9 (34.6) | 0.333 | 4 (25.0) | 5 (31.3) | 0.141 |
| HLA-haploidentical, n (%) | 17 (68.0) | 23 (88.5) | 0.502 | 12 (75.0) | 13 (81.3) | 0.154 |
| ABO mismatch, n (%) | 9 (36.0) | 15 (57.7) | 0.437 | 7 (43.8) | 8 (50.0) | 0.125 |
| Mean SMD | — | — | 0.335 | — | — | 0.080 |

Abbreviations: SMD, standardized mean difference; IST, immunosuppressive therapy; EBMT, European Society for Blood and Marrow Transplantation.

**Supplementary Table S2. Baseline characteristics after stabilized inverse-probability-of-treatment weighting (IPTW), full cohort n=51**

Continuous variables presented as weighted median; categorical variables as weighted percentage. SMD < 0.1 represents satisfactory balance.

| **Covariate** | **FMA(weighted n=24.7)** | **FCA(weighted n=26.3)** | **SMD after stabilized-IPTW** |
| --- | --- | --- | --- |
| Age at transplantation, years | 15.3 | 14.7 | 0.063 |
| Sex, female (%) | 48.9 | 46.2 | 0.054 |
| Diagnosis VSAA (%) | 17.1 | 19.4 | 0.058 |
| Pre-transplant IST-Yes (%) | 51.4 | 52.7 | 0.026 |
| EBMT score ≥2 (%) | 54.8 | 53.1 | 0.034 |
| Diagnosis-to-transplant ≥1y (%) | 28.3 | 30.5 | 0.047 |
| HLA-haploidentical (%) | 79.2 | 77.6 | 0.038 |
| ABO mismatch (%) | 47.1 | 49.3 | 0.043 |
| Mean SMD | — | — | 0.072 |

**Supplementary Table S3. Summary of survival analyses based on PSM-matched cohort and stabilized-IPTW full cohort**

OS: overall survival; HR: hazard ratio; CI: confidence interval.

| **Analysis setting** | **Group** | **n** | **3-year OS** | **Log-rank P** | **HR (FMA vs FCA)** | **95% CI for HR** | **P value** | **PH-assumption(Schoenfeld P)** |
| --- | --- | --- | --- | --- | --- | --- | --- | --- |
| 1:1 PSM-matched cohort | FMA | 16 | 93.8% | 0.156 | 0.236 | 0.026-2.115 | 0.197 | 0.106 |
|  | FCA | 16 | 75.0% |  |  |  |  |  |
| Stabilized-IPTW full cohort | FMA | 25 | 91.2% | 0.118 | 0.271 | 0.061-1.204 | 0.087 | 0.121 |
|  | FCA | 26 | 70.4% |  |  |  |  |  |

PSM: 1:1 optimal propensity-score matching, caliper = 0.40 on logit-PS scale.

Stabilized-IPTW: stabilized inverse-probability-of-treatment weighting including all 51 FMA/FCA patients.

Proportional-hazards (PH) assumption was assessed using Schoenfeld residual test; P > 0.05 indicates PH assumption holds.

No statistically significant survival difference was identified in either analysis; consistent HR < 1 indicated only a numerical trend toward better survival for FMA.
